# Supplementary material for: Uncovering the SUMOylation and ubiquitylation crosstalk in human cells using sequential peptide immunopurification
Source: Nat Commun. 2017 Jan 18;8:14109. doi: 10.1038/ncomms14109 (PMC5253644; doi:10.1038/ncomms14109)
Supplement: Supplementary Information — Supplementary Figures and Supplementary References [file ncomms14109-s1.pdf]

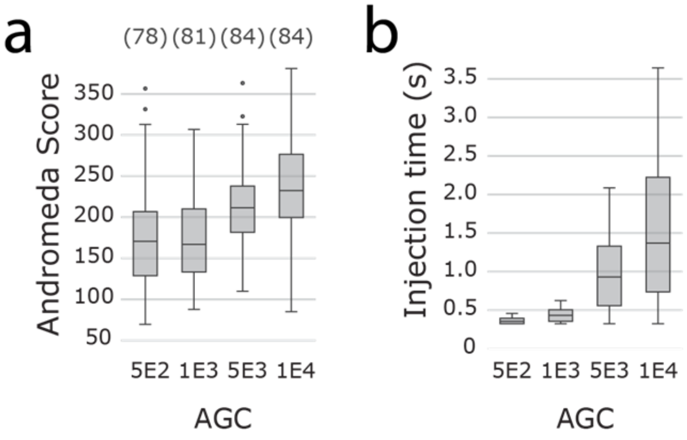

Supplementary Figure 1 - MS Optimization: (a) Distribution of the Andromeda scores of synthetic SUMOylated peptides identified with various AGC target values (5E2, 1E3, 5E3, 1E4). The number above the box plot represents the number of identified peptides. Error bars correspond to the standard deviation from instrumental triplicates. (b) Distribution of injection times required to reach variable target AGC value (5E2, 1E3, 5E3, 1E4) based on the samples in a.

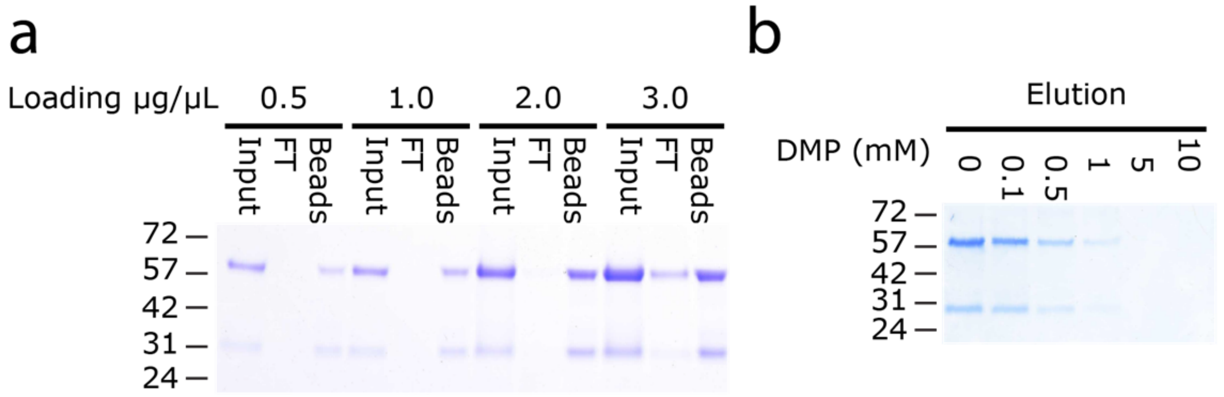

Supplementary Figure 2 - Preparation of the Anti-K-(NQTGG) Bound Protein A/G Beads: (a) Determining the loading capacity of the Protein A/G beads for the anti-K-(NQTGG) antibody. Coomassie-stained gel of variable amounts of antibody (5, 10, 20 and 30 µg) incubated with 10 µL of protein A/G bead slurry. (b) Colloidal Coomassie-stained gel of 10 µL of saturated bead slurry (20 µg of antibody) cross-linked with variable concentrations of DMP (100 µL at 0.1, 0.5, 2, 5 and 10 mM).

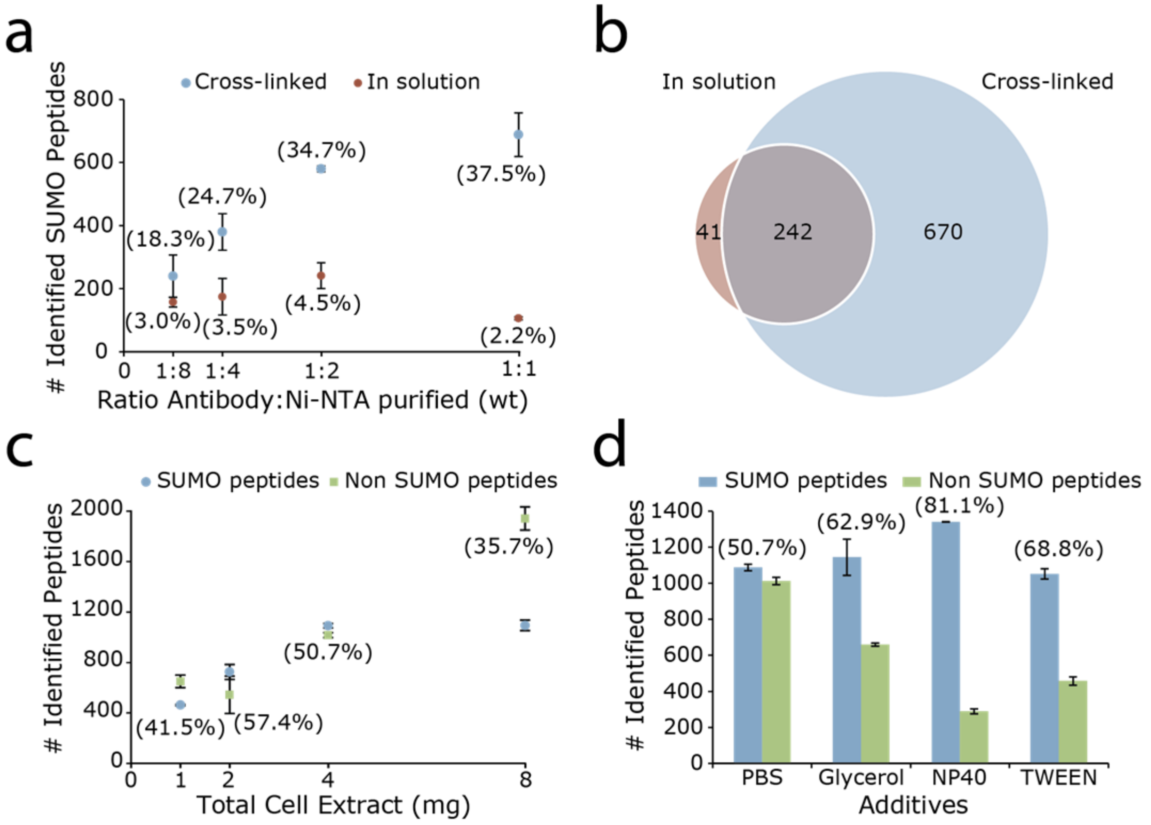

Supplementary Figure 3 – Anti-K-(NQTGG) IP Optimization: (a) Optimization of the antibody:NiNTA purified material ratio using a 1:8, 1:4; 1:2 and 1:1 of AB:TCE with in solution (red) or cross-linked (blue) antibody. The number above the error bars corresponds to the SUMO enrichment level. (b) Overlap of all the identified SUMOylated peptides using the in solution (red) or cross-linked (blue) antibody. (c) Optimization of the starting material. Titration curve of SUMOylated peptides (blue) and non SUMOylated peptides (green) identified after immunopurification with a fixed ratio Antibody:NiNTA purified material (1:2) and increasing amounts of total cell extract (1, 2, 4 and 8 mg). The number above the error bars corresponds to the SUMO enrichment level. (d) Optimization of the buffer composition for SUMO IP. SUMOylated peptides (blue bars) and non SUMOylated peptides (green bars) identified after immunopurification with a fixed ratio Antibody:NiNTA purified material (1:2) and amount of material (4 mg) with different buffers (PBS, PBS + 50% glycerol, PBS + 0.1% NP40 and PBS + 0.1% TWEEN). Error bars correspond to the standard deviation from technical replicates starting from the same NiNTA enriched material.

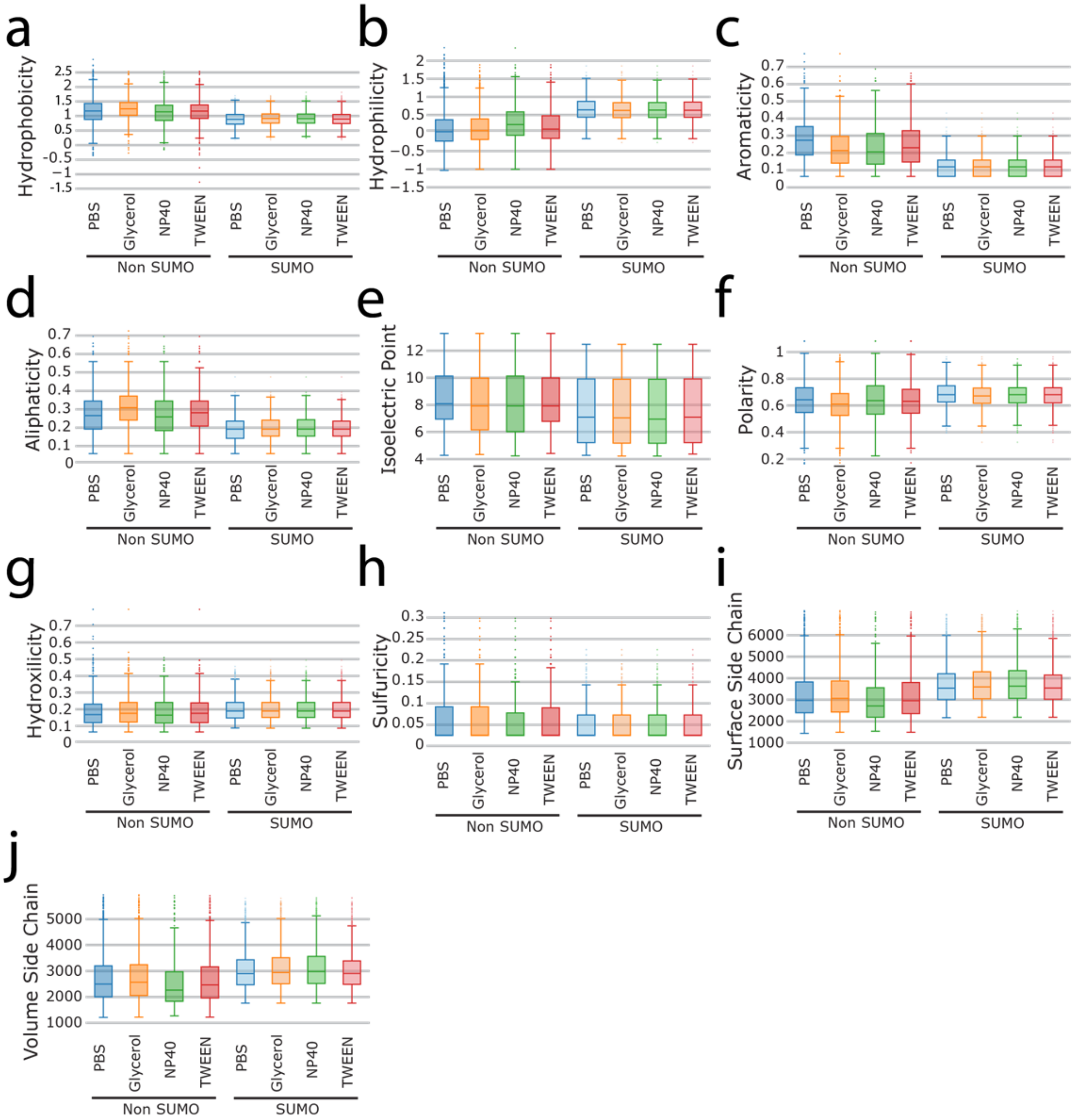

Supplementary Figure 4 - Physiochemical Properties of SUMO and Non SUMO Peptides: Distribution of Hydrophobicity (a), Hydrophillicity (b), Aromaticity (c), Alliphaticity (d), Isoelectric Point (e), Polarity (f) Hydroxyllicity (g), Sulfuricity (h) Surface of the Side Chain (i) and Volume of the Side Chain (j) of the identified non SUMOylated and SUMOylated peptides retained after IP using various incubation buffers.

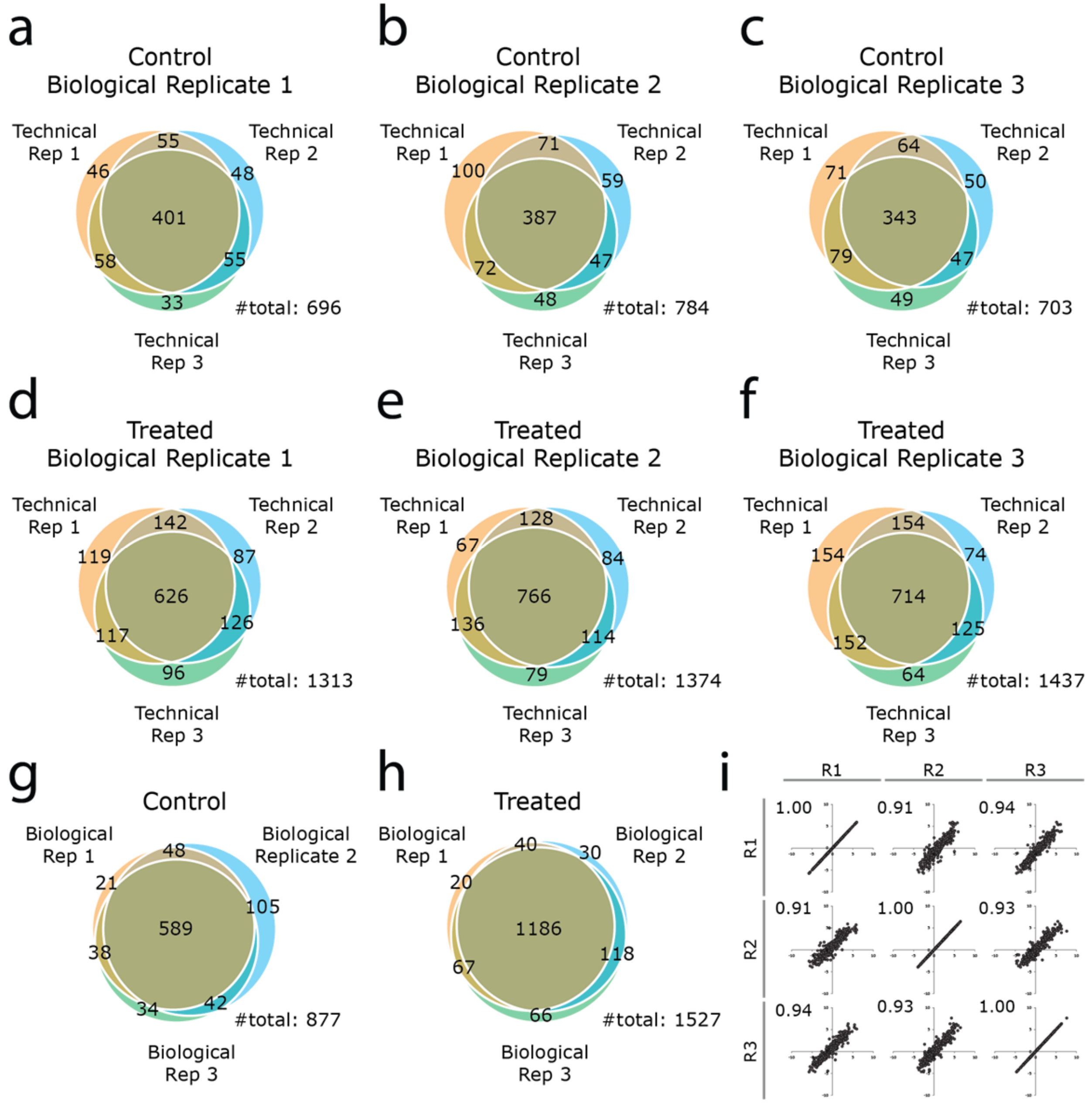

Supplementary Figure 5 – SUMO Peptide Purification Workflow Reproducibility: Overlap of identified SUMOylated peptides across three technical replicates for control (a, b and c) and MG132-treated (d, e and f) samples. Overlap of identified peptides for three biological samples containing three technical replicates for control (g) and MG132-treated (h) samples. (i) Reproducibility in fold changes of SUMOylated peptide intensities across three biological replicates of MG132 and control cells.

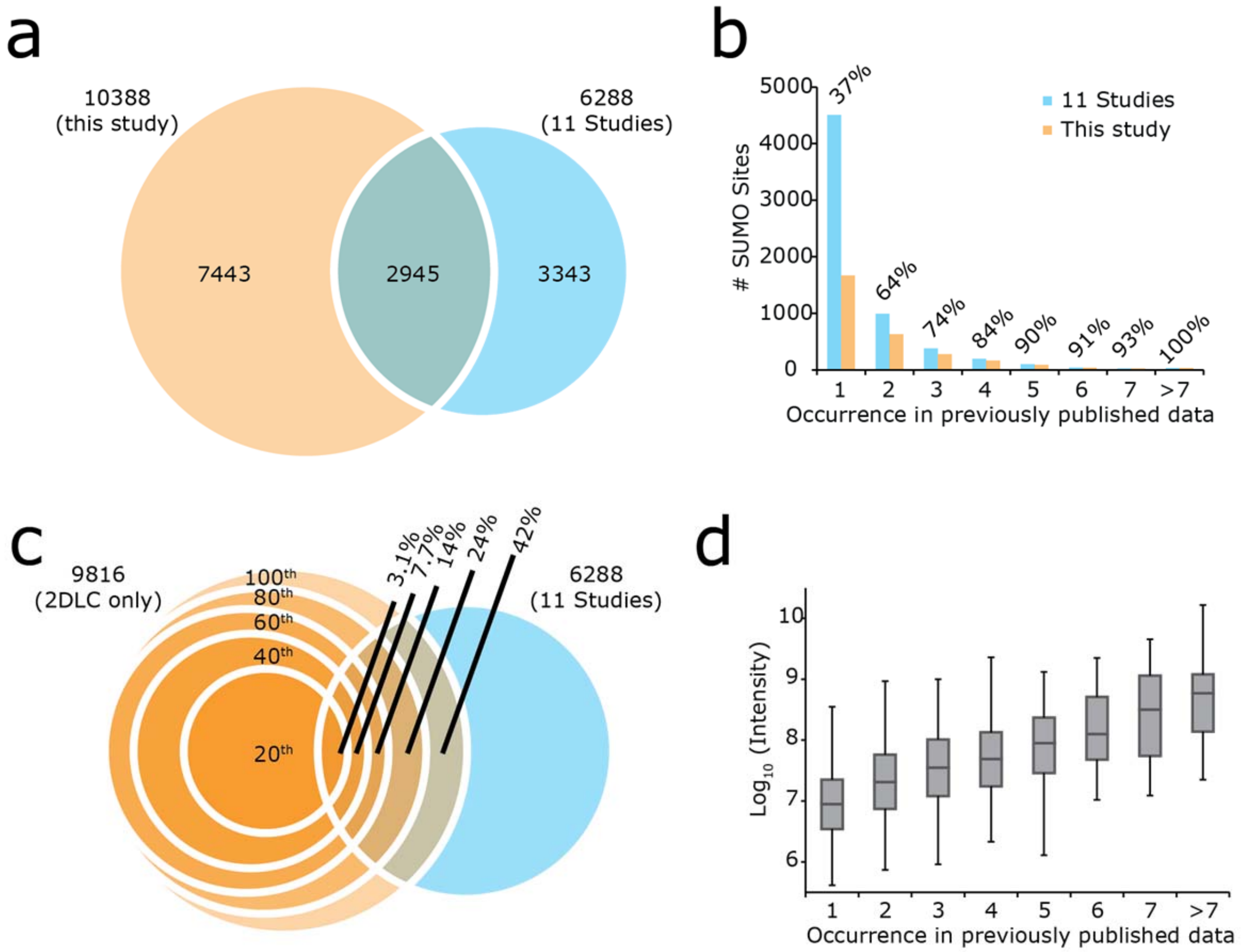

Supplementary Figure 6 - Comparison of the SUMO Sites Identified in this Study to those Reported to Date:

(a) Overlap of the SUMO sites identified in this study to those documented in the literature [1-11]. (b) Occurrence of previously identified SUMOylation sites in the 11 studies. The bleu bars represent the compilation of 11 dataset and the orange bars this study. The values above the bars represent the proportion of the 11 datasets, which were identified in the current study. (c) Overlap of the identified SUMOylation sites obtained from the 2D-LC experiment (orange circle) to those identified in the 11 studies (blue circle). Orange circles are divided in intensity percentiles using a bin of 20%, where the smaller orange circle represents the 20% least intense SUMOylation sites. (d) Box plot representation of the peptide intensities of the SUMOylated peptides identified in this study with respect to the number of times the SUMOylation sites have been reported in the 11 datasets.

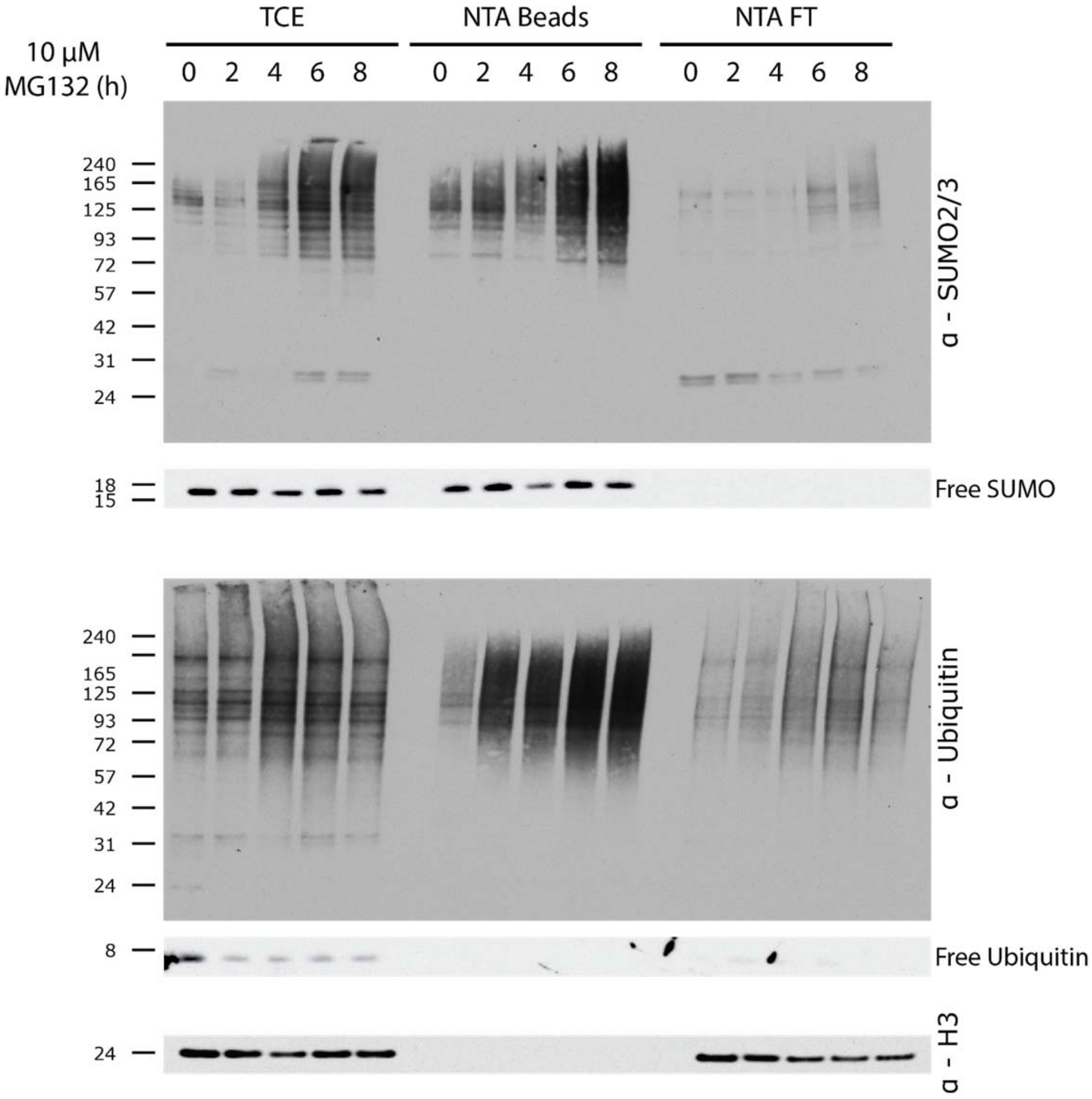

Supplementary Figure 7 – Temporal Profiling of SUMO and Ubiquitin Changes in Response to MG132

Western blots of total SUMO and Ubiquitin levels in response to increasing MG132 treatment times.

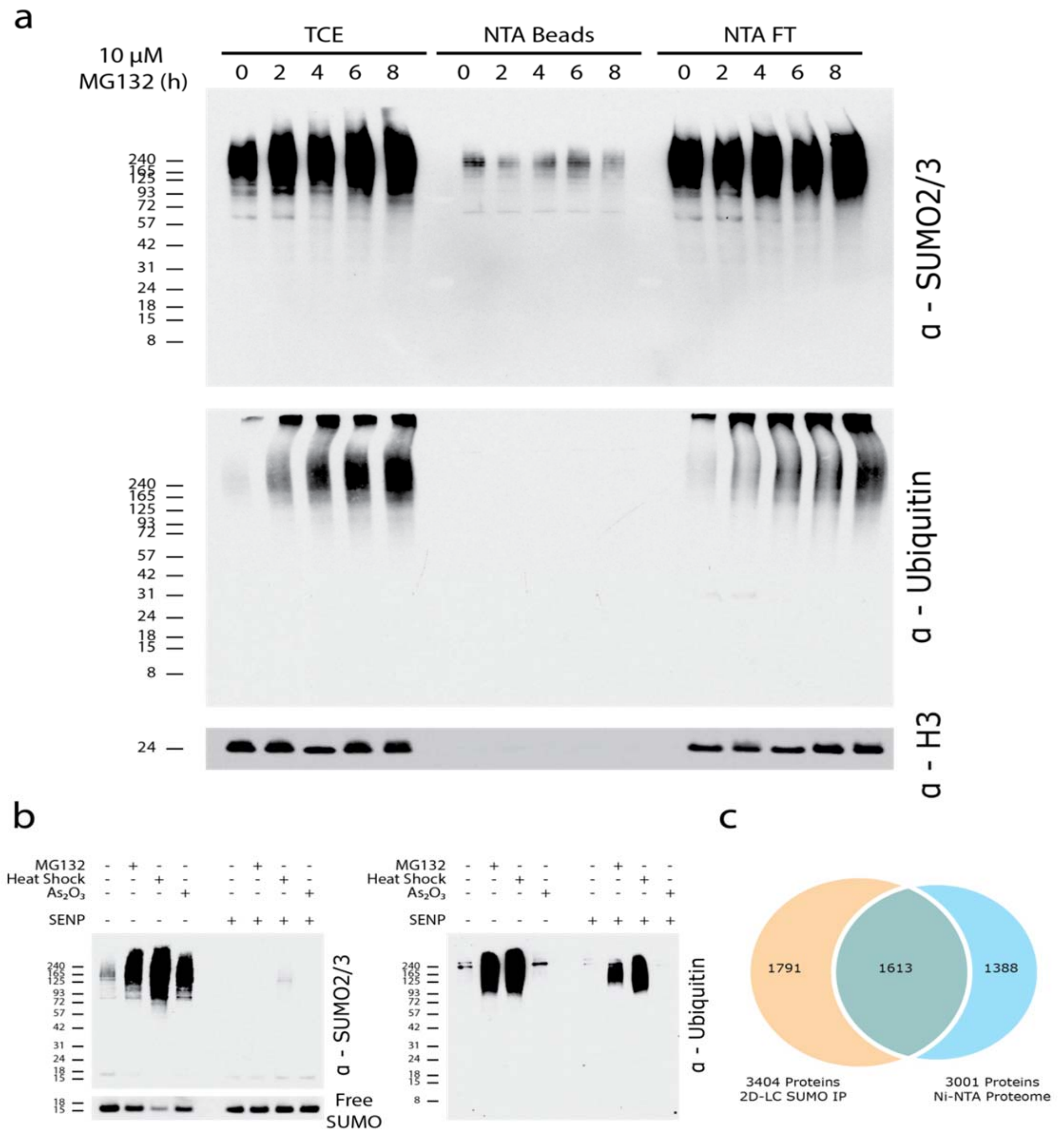

Supplementary Figure 8 – Specificity of Ni-NTA Purification Step: (a) Western blot of NiNTA enriched proteins from HEK293 cells upon prolonged exposure to MG132 (10 µM). (b) Western blot of NiNTA enriched proteins from SUMO3m expressing cells untreated and treated with MG132, Heat shock or As_2O_3. Enriched extracts subjected to SENP proteolysis (+SENP) showed the absence of SUMO signals at higher masses while the ubiquitin signal distribution showed a reduction in molecular weight suggesting that the ubiquitylated proteins in the NiNTA enriched samples were also SUMOylated. (c) Venn diagram of proteins identified as SUMOylated in this study and the proteins identified after the NiNTA purification.

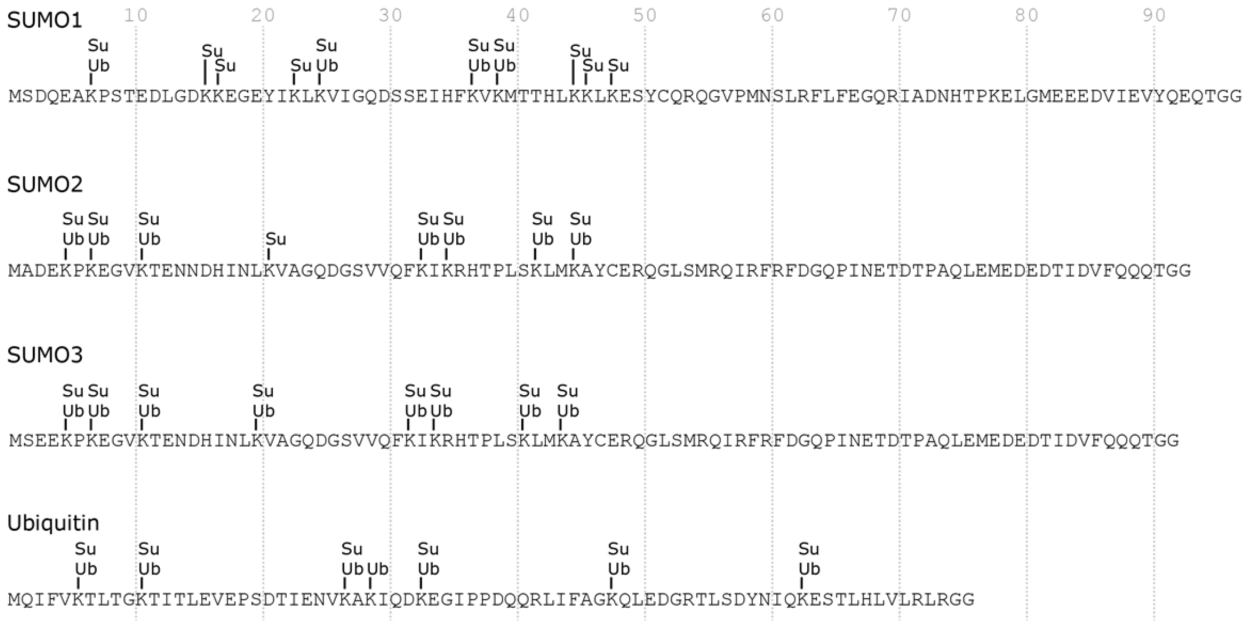

Supplementary Figure 9 – Mapped Residues Involved in PolySUMO and PolyUbiquitin Chain Formation: List of all the SUMOylation and Ubiquitylation branching sites identified on SUMO1, 2, 3 and Ubiquitin in this study.

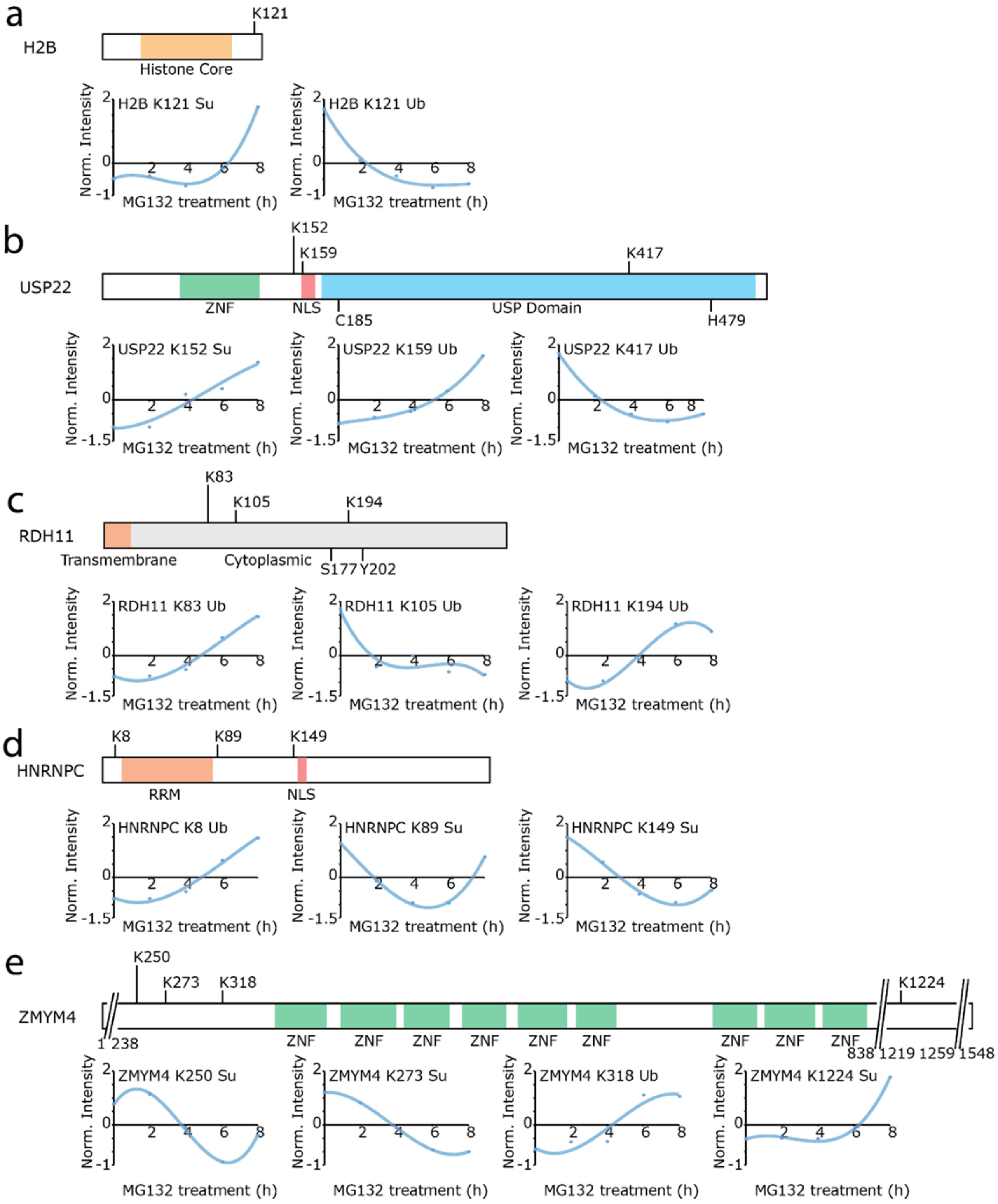

Supplementary Figure 10 – List of Proteins Displaying Bidirectional Kinetic Profiles of SUMO and/or Ubiquitin Levels in Response to MG132 Treatment: Cartoon representation of protein substrates and the kinetic profiles associated with Ubiquitin and SUMO levels at each profiled site. (a) H2B, (b) USP22, (c) RDH11, (d) HNRNPC, and (e) ZMYM4.

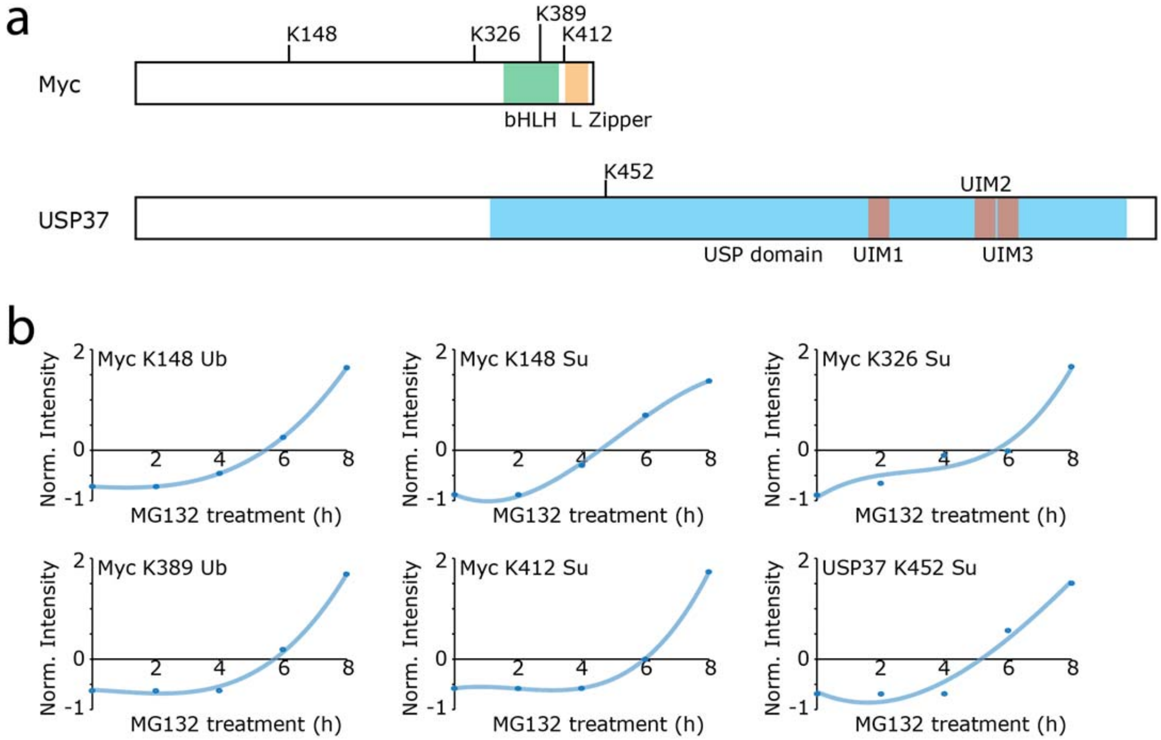

Supplementary Figure 11 – Role of USP37 SUMOylation on the Levels of Myc Ubiquitylation and SUMOylation: (a) Cartoon representation of USP37 and Myc domains with the profiled SUMO and Ubiquitin sites annotated. (b) Kinetic profiles of the SUMO and Ubiquitin modified sites identified on USP37 and Myc.

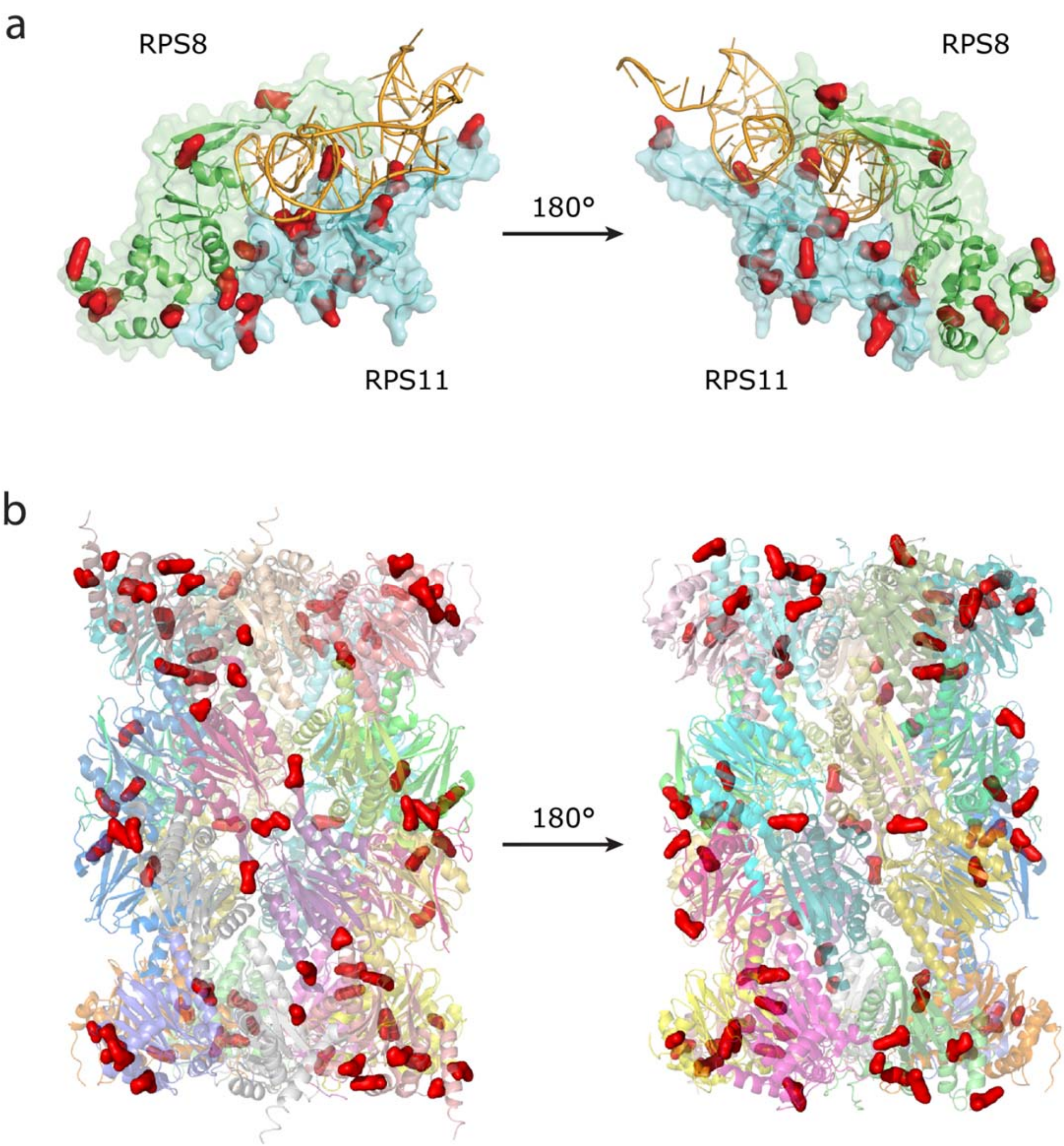

Supplementary Figure 12 – 3D Mapping of the Modified Lysine Residue Identified on the Ribosome and the Proteasome: (a) Ribosomal complex between RSP8 (green), RSP11 (blue) and the strand of RNAr (orange) (extracted from PDB: 4UG0). Modified lysine residues are represented in red. (b) Crystal structure of the 20S proteasome (PDB:4R3O) with all the modification sites identified in both the kinetic and 2D-LC experiments mapped in red. Only 5 sites are located in the complex lumen while 36 are present at the surface of the protein complex.

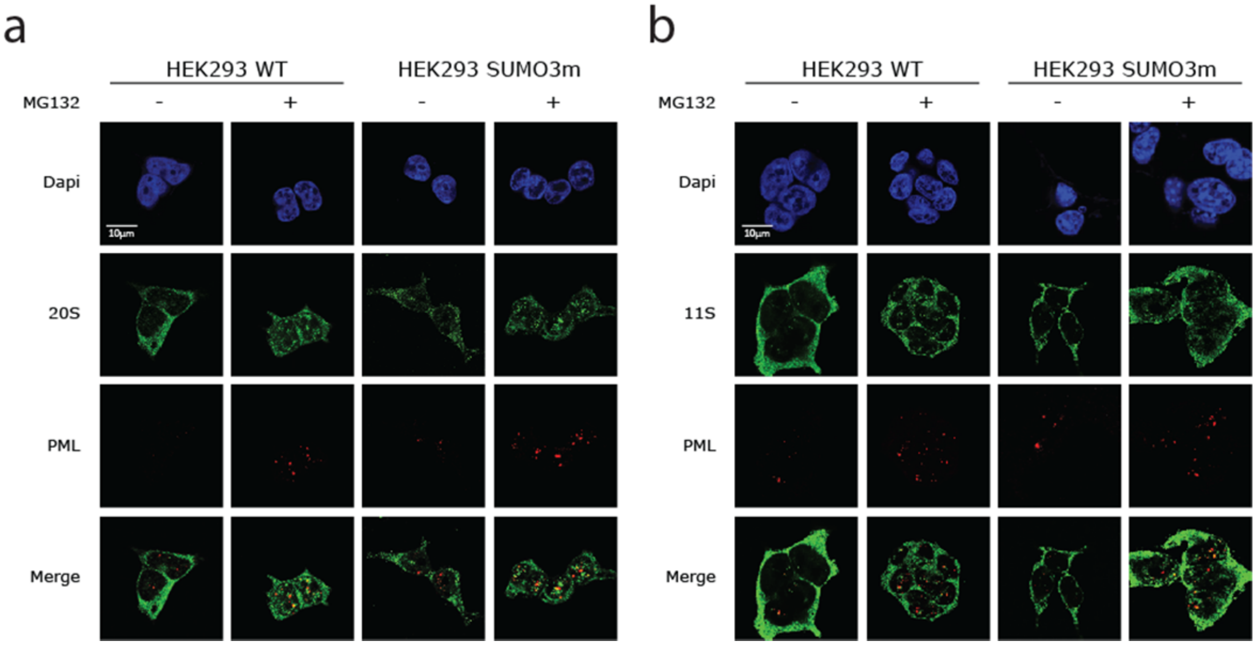

Supplementary Figure 13 – Immunofluorescence of 20S and 11S Proteasome and PML under MG132 stress: HEK293 WT and HEK293-SUMO3m cells were treated with 10 µM MG132 for 6 h. Double immunofluorescence analyses were performed using monoclonal anti-PML (red) and (a) rabbit anti-20S (green) or (b) rabbit anti-11S (green) antibodies.

References

1. Lamoliatte, F. et al. Large-scale analysis of lysine SUMOylation by SUMO remnant immunoaffinity profiling. *Nature Communications* **5**, 5409 (2014).
2. Tammsalu, T. et al. Proteome-wide identification of SUMO2 modification sites. *Science Signaling* **7**, rs2 (2014).
3. Impens, F., Radoshevich, L., Cossart, P. & Ribet, D. Mapping of SUMO sites and analysis of SUMOylation changes induced by external stimuli. *Proceedings of the National Academy of Sciences of the United States of America* **111**, 12432-12437 (2014).
4. Hendriks, I.A. et al. Uncovering global SUMOylation signaling networks in a site-specific manner. *Nature Structural & Molecular Biology* **21**, 927-936 (2014).
5. Hendriks, I.A., D'Souza, R.C., Chang, J.-G., Mann, M. & Vertegaal, A.C.O. System-wide identification of wild-type SUMO-2 conjugation sites. *Nature Communications* **6**, 7289 (2015).
6. Bursomanno, S. et al. Proteome-wide analysis of SUMO2 targets in response to pathological DNA replication stress in human cells. *DNA repair* **25**, 84-96 (2015).
7. Hendriks, I.A., Treffers, L.W., Verlaan-de Vries, M., Olsen, J.V. & Vertegaal, A.C.O. SUMO-2 Orchestrates Chromatin Modifiers in Response to DNA Damage. *Cell Reports* (2015).
8. Matic, I. et al. Site-specific identification of SUMO-2 targets in cells reveals an inverted SUMOylation motif and a hydrophobic cluster SUMOylation motif. *Molecular Cell* **39**, 641-652 (2010).
9. Schimmel, J. et al. Uncovering SUMOylation dynamics during cell-cycle progression reveals FoxM1 as a key mitotic SUMO target protein. *Molecular Cell* **53**, 1053-1066 (2014).
10. Sohn, S.-Y., Bridges, R.G. & Hearing, P. Proteomic analysis of ubiquitin-like posttranslational modifications induced by the adenovirus E4-ORF3 protein. *Journal of Virology* **89**, 1744-1755 (2015).
11. Xiao, Z. et al. System-wide Analysis of SUMOylation Dynamics in Response to Replication Stress Reveals Novel Small Ubiquitin-like Modified Target Proteins and Acceptor Lysines Relevant for Genome Stability. *Molecular & cellular proteomics: MCP* **14**, 1419-1434 (2015).
